# Prognosis in Lobar Pneumonia

**Published:** 1907-03-23

**Authors:** 


### PROGNOSIS IN LOBAR PNEUMONIA.

At a meeting of the Medico-Chirurgical Society of Edinburgh, on March 6th, the President, Dr. J. O. Affleck, read a paper detailing his experience in the prognosis of lobar pneumonia. Though no statistics were available, Dr. Affleck stated his belief that pneumonia had not only become more prevalent, but had assumed a more serious character in recent years. The mortality from the disease had risen during the last sixteen years, probably from the prevalence of influenza during that period. Not only did influenza predispose to pneumonia, but by subjecting the patient to the influence of a double toxæmia it rendered his condition much more serious. The mortality of such influenzal pneumonias had been very high. Prognosis in pneumonia was a matter of great difficulty, as no one could say at the outset how any given case might end. Excluding infancy, the youth of the patient was a most favourable factor, as above the age of fifty the prognosis was grave, however healthy the patient. Alcoholism was a serious complication, and was sufficient to counter-balance all the advantages of youth and vigour. Chronic pre-existing conditions, such as Bright's disease or the presence of the specific fevers, affected the prognosis, and pneumonia was also very fatal in the insane. After operations the occurrence of pneumonia is a serious complication, but not invariably a fatal one.

The mode of onset may give some aid in prognosis, as in the case where the patient is obviously overwhelmed by the toxæmia from the first, and death takes place at an early stage. Cases which exhibit gastric or abdominal symptoms or which simulate lesions of the abdominal viscera are frequently serious. Where there is copious perspiration along with high temperature at the outset, the prognosis is grave. The diagnosis is apt to be obscured by nervous symptoms at the onset, as by convulsions in childhood, which are not serious; but the occurrence of delirium tremens is a sign of the gravest omen.

During the course of the disease persistent insomnia, where drugs fail to induce sleep, is a sign of danger, as is the occurrence of delirium, whether of the active or low typhoid form. The temperature is not a reliable guide to prognosis in pneumonia. It is best when highest at the onset and falls slightly towards the crisis, but a pseudo-crisis may occur about the fifth day, and be followed by a rise. A persistent high temperature, *e.g.*, 105° F., without remissions indicates a severe type of the disease, while in the aged a low temperature is an indication of gravity.

The condition of the heart is the main determining factor in the prognosis of pneumonia. While the dangers from overtasking the right side of the heart are well recognised, the state of the left ventricle must not be forgotten, and both sudden and gradual heart-failure are of common occurrence. The pulse-rate in pneumonia is usually about 120, and when this is not exceeded the prognosis is favourable. Should the pulse exceed 130 the prognosis is unfavourable, and when 140 is reached the case is of the utmost gravity. A change in character of the pulse—*e.g.*, when it becomes small and rapid—usually indicates the approach of a fatal issue. In the aged the rate of the pulse is less important than its character. A moderate amount of expectoration is usual and is most favourable when of a rusty colour rather than when more deeply bloodstained, but the latter is less ominous than the occurrence of prune-juice sputum. The cessation of expectoration with increasing cyanosis is an ominous sign. While within limits the amount of consolidation does not directly affect the issue, the complete consolidation of one lung or the implication of both lungs materially increases the gravity of the prognosis.

Any irregularity in the occurrence of the crisis is usually of an unfavourable nature. Should the temperature fall while the pulse continues rapid, there is danger of heart-failure. A rise of temperature after the crisis should suggest an extension of the pneumonic process or the occurrence of empyema, a not uncommon complication of influenzal pneumonia. Any sign of syncope or exhaustion about the crisis is a sign of danger. Lysis is chiefly met with in alcoholic subjects, and in insidious cases, which latter often run an irregular course.

